# Supplementary figures and images for: Blunted diurnal firing in lateral habenula projections to dorsal raphe nucleus and delayed photoentrainment in stress-susceptible mice
Source: PLoS Biol. 2021 Mar 10;19(3):e3000709. doi: 10.1371/journal.pbio.3000709 (PMC7984642; doi:10.1371/journal.pbio.3000709)

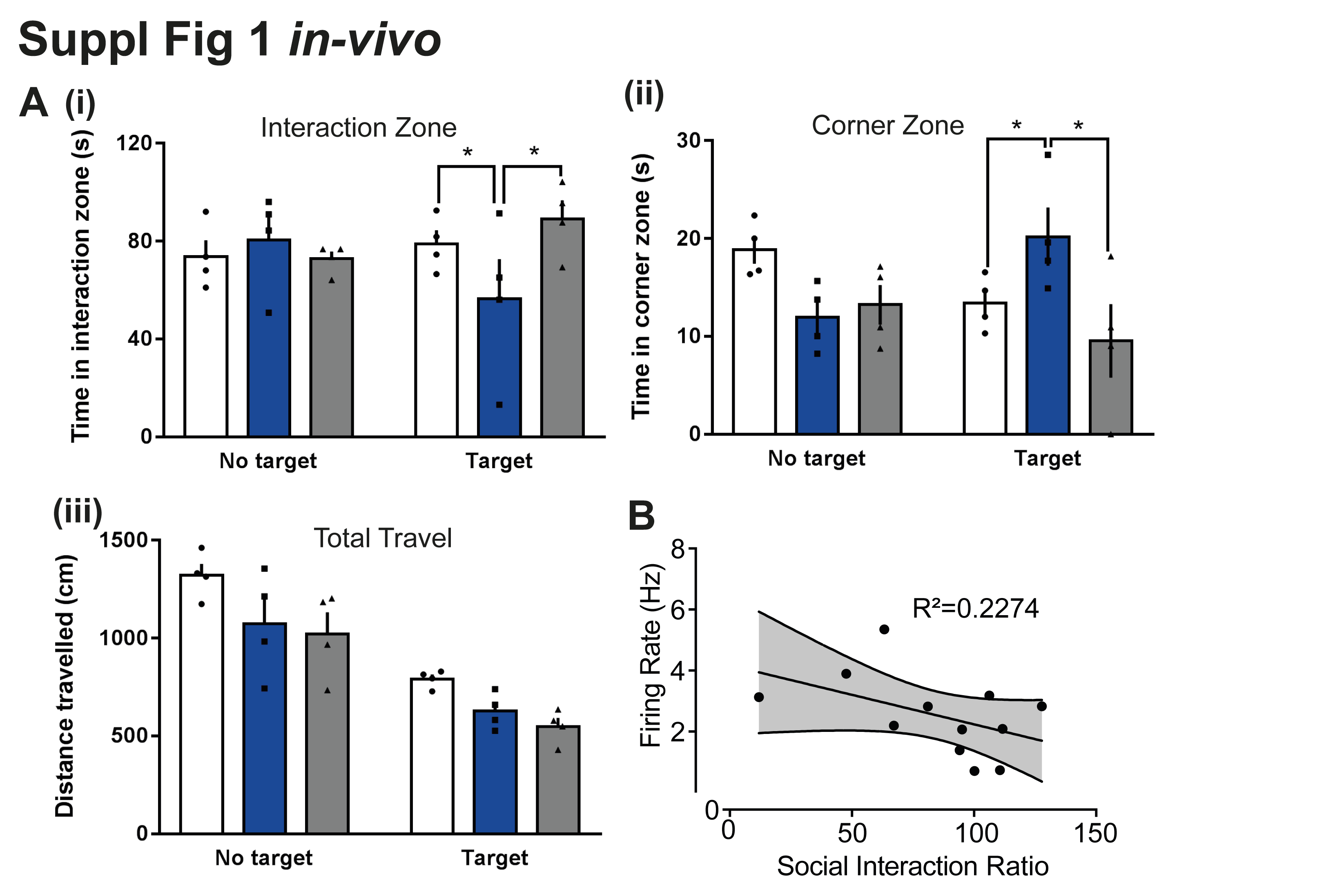

Supplement: S1 Fig — Social stress induces social avoidance in susceptible mice. (A) SI data showed that in the presence of a CD1 social target (nonaggressor), susceptible mice displaying: (i) decreased time in the interaction zone (F2,9 = 5.185, P < 0.05), (ii) increased time in the corner zone (F2,9 = 5.188, P < 0.05), and (iii) no difference in total travel between control, susceptible, and resilient mice. (B) Though not significant, correlation analysis of in vivo spontaneous activity in the LHb and SI ratio showed a very slight negative correlation. Error bars: mean ± SEM. The raw data can be found in S7 Data. LHb, lateral habenula; SI, social interaction. (TIF) [file pbio.3000709.s001.tif]

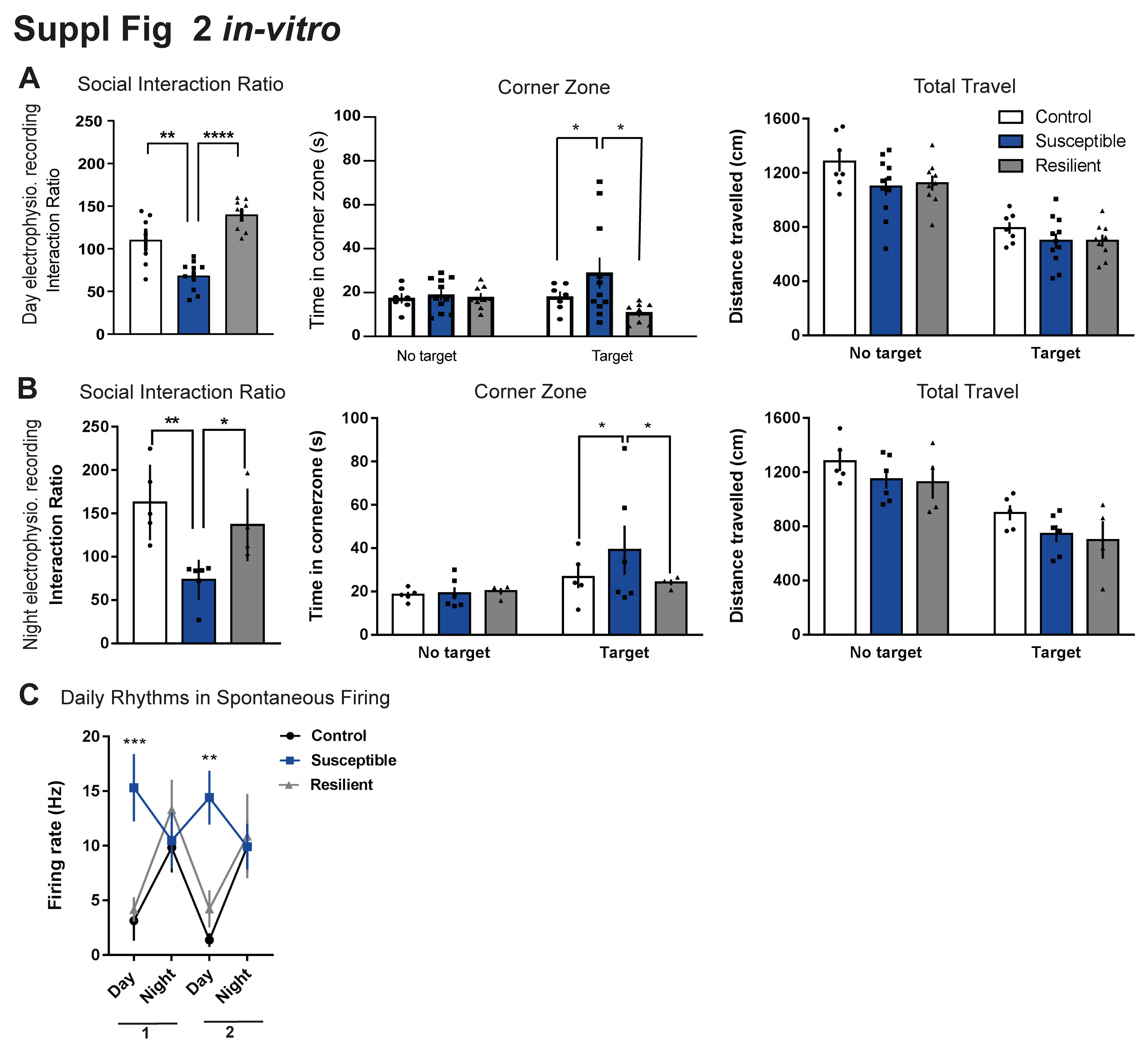

Supplement: S2 Fig — SI data in mice used to measure day or nighttime spontaneous firing in cellsLHb→DRN. (A) Susceptible mice used for day or (B) nighttime in vitro recording of labelled cellsLHb-DRN display: decreased SI ratio (day—F2,24 = 28.02, P < 0.0001; n = 7–11 mice/group; night—F2,12 = 8.959, P < 0.01; n = 4–6 mice/group) (left). In the presence of CD1 (social target) susceptible mice spent increased time in the corner zone (day—F2,24 = 3.335, P < 0.05; night—F2,12 = 6.444, P < 0.05) (middle). There was no difference in total travel between control, susceptible, and resilient mice (right). (C) The in vitro electrophysiology experiments were performed over 16 days after the SI test. To better visualize the rhythmic changes in spontaneous firing in cellsLHb→DRN, data were regraphed where day and night firing was binned into first half (1–8 Day/Night after SI) and the second half (9–16 Day/Night after SI) recording sessions. In susceptible mice, the day time firing pattern is phase inversed and was significantly higher on day 1 than control and resilient mice (Day 1 –control vs susceptible: F2,160 = 3.617, P = 0.0306; resilient vs susceptible: F2,160 = 3.689, P = 0.0268; n = 53–64 cells from 7 to 11 mice/group), and day 2 from control mice (Day 2 –F2,160 = 3.653, P = 0.0286), ***day vs day in control vs susceptible and resilient vs susceptible mice, **control vs susceptible. Error bars: mean ± SEM. The raw data can be found in S8 Data. SI, social interaction. (TIF) [file pbio.3000709.s002.tif]

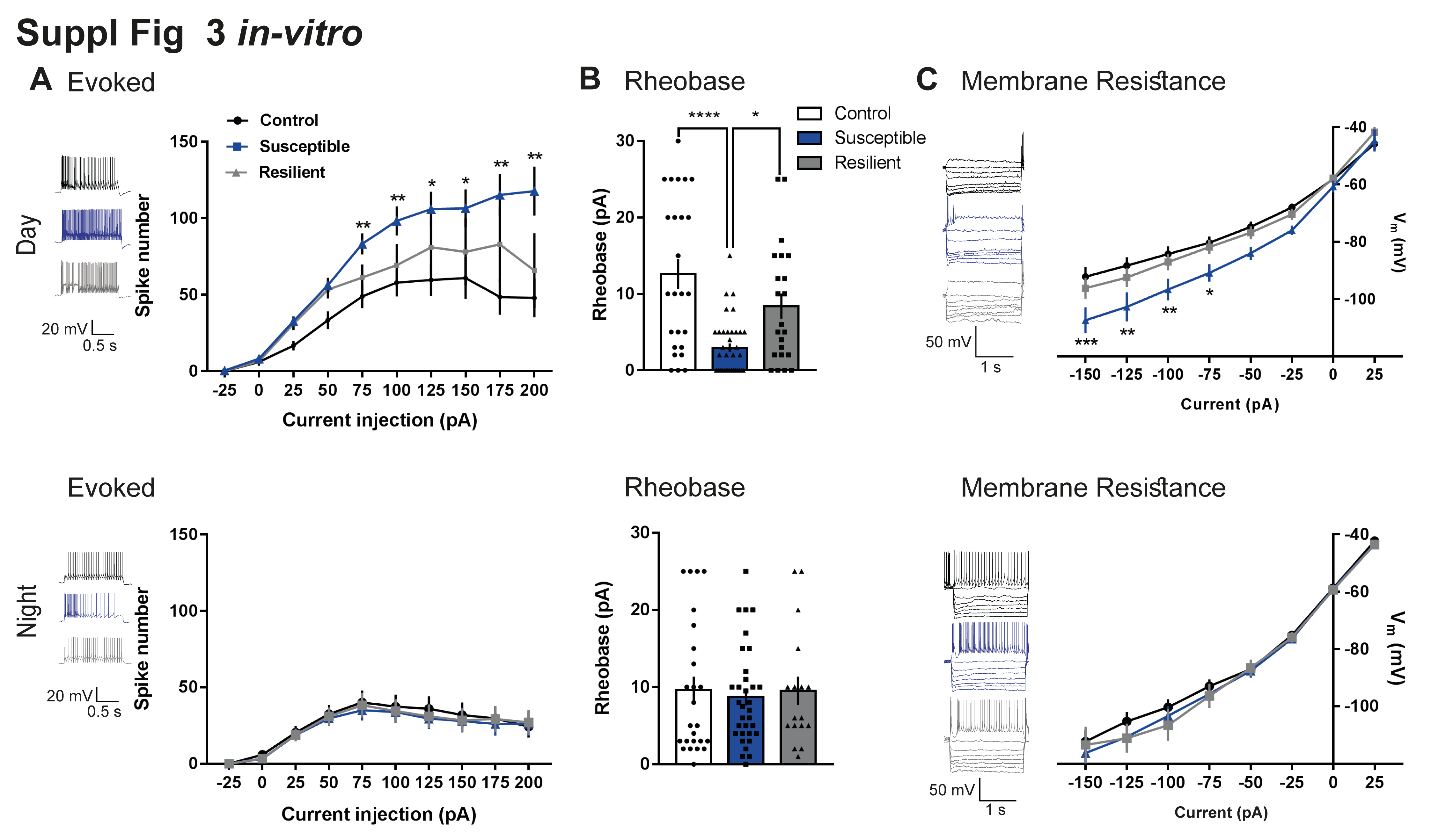

Supplement: S3 Fig — Diurnal difference in intrinsic membrane properties in mice exposed to CSDS. (A) Sample traces of evoked firings of cellsLHb-DRN in day (top left) or night (bottom left) in control (top), susceptible (middle), and resilient mice (bottom). CellsLHb-DRN from susceptible mice display increased daytime excitability in response to incremental steps in current injections (75, 100, 125, 150, 175, and 200 pA) compared with control and resilient mice (F2,820 = 21.61, P < 0.0001; n = 14 to 42 cells from 7 to 11 mice per group) (top right). There was no difference between the stress phenotypes at night (bottom right). (B) CellsLHb-DRN from susceptible mice display decreased daytime rheobase (F2,82 = 14.37, P < 0.0001; n = 22 to 38 cells from 7 to 11 mice per group) (top). There was no difference in nighttime rheobase between the stress phenotypes (bottom). (C) Representative voltage traces in response to hyperpolarizing current injections in cellsLHb-DRN in day (top left) or night (bottom left) in control (top), susceptible (middle), and resilient mice (bottom). Daytime I–V relationship showing cellsLHb-DRN from susceptible mice display increased membrane resistance (F2,264 = 23.13, P < 0.0001; n = 9 to 15 cells from 7 to 11 mice per group) (top right). There was no difference in Nighttime I–V relationship between the stress phenotypes (bottom right). Error bars: mean ± SEM. The raw data can be found in S9 Data. CSDS, chronic social defeat stress. (TIF) [file pbio.3000709.s003.tif]

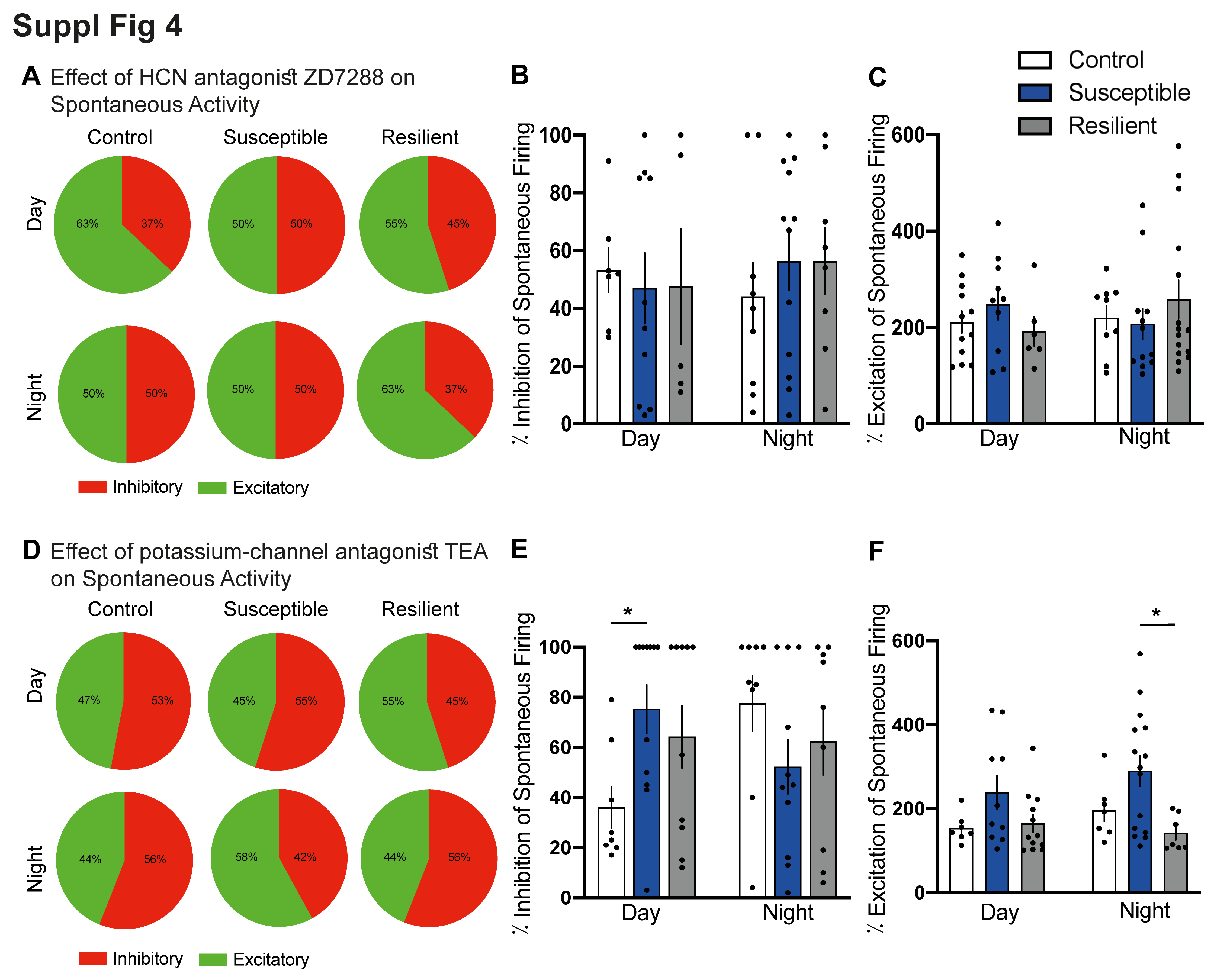

Supplement: S4 Fig — Effects of HCN channel and KV+-channel antagonists on spontaneous activity. (A) Pie charts illustrating the percentage of cells excited or inhibited by HCN antagonist ZD7288 (10 μM). (B, C) One-way ANOVA does not show significant difference in the % excitation or inhibition of spontaneous firing between control, resilient, and susceptible mice in the day or the night. (Day % Inhibition: n = 5–10 cells; Day % Excitation: n = 6–12 cells; Night % Inhibition: n = 8–12 cells; Night % Excitation: n = 9–15 cells from 2–3 mice/group). (D) Pie charts illustrating the percentage of cells excited or inhibited by KV+-channel antagonist TEA (10 mM). (E) One-way ANOVA shows significant difference in the % inhibition in the day (F2,27 = 3.42, P = 0.047, n = 8–12 cells from 2–3 animals/group). Post hoc analysis showed significant % inhibition in susceptible mice compared to control mice (Tuckey multiple comparison: P = 0.001). There was no significant difference in % inhibition at night (n = 9–11 cells from 2–3 animals/group). (F) One-way ANOVA shows significant difference in % excitation in the night (F2,26 = 4.63, P = 0.02, n = 7–15 cells from 2–3 animals/group). Post hoc analysis showed significant % excitation in susceptible mice compared to resilient mice (Tuckey multiple comparison: P = 0.02). There was no statistical difference in % excitation in the day (n = 7–12 cells from 2–3 animals/group). The raw data can be found in S10 Data. HCN, hyperpolarization-activated cation; TEA, tetraethylammonium. (TIF) [file pbio.3000709.s004.tif]

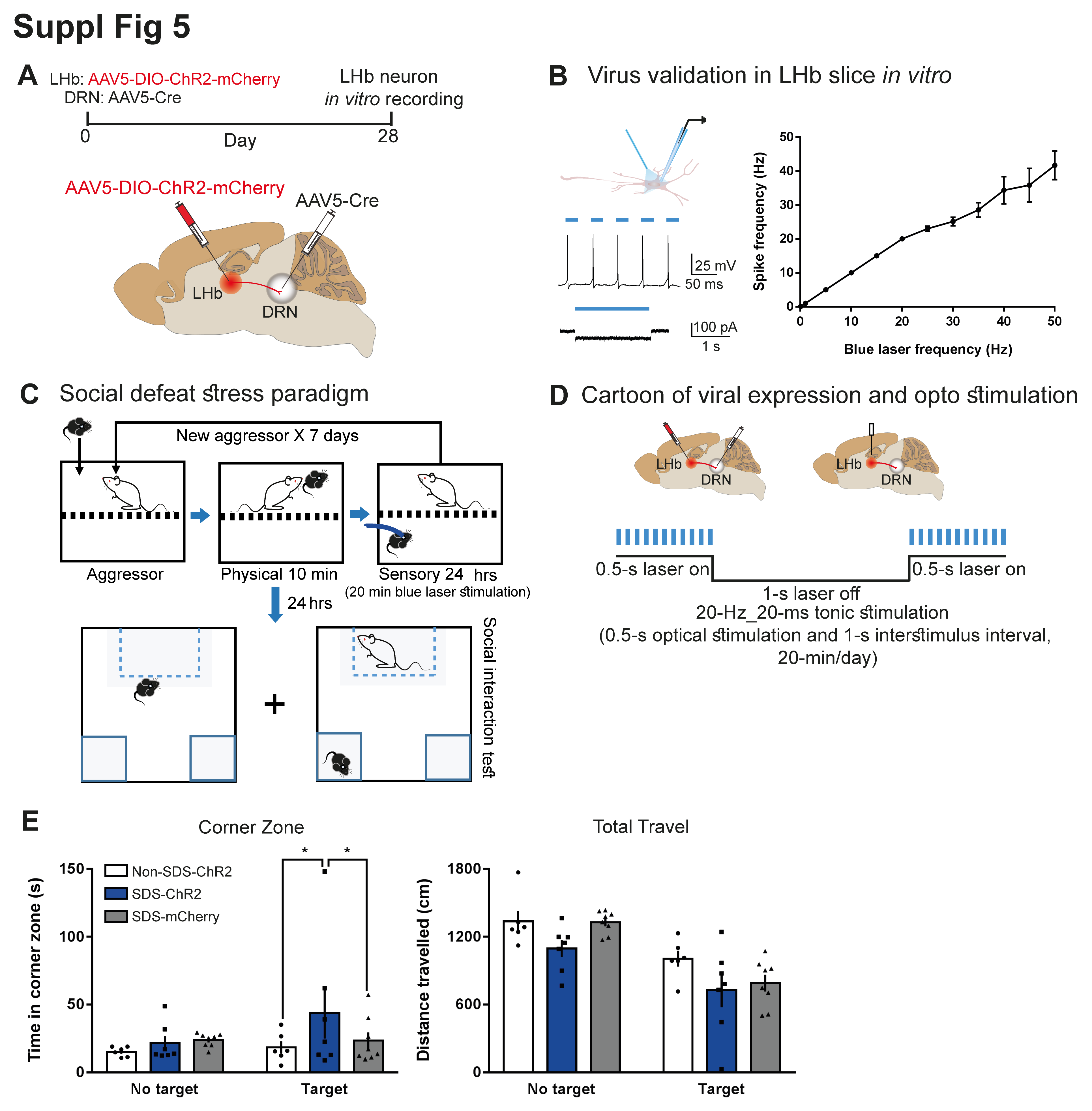

Supplement: S5 Fig — Functional validation of AAV-DIO-ChR2-mCherry and optical stimulation after each exposure to social stress during the SDS paradigm. (A) Experimental timeline of pathway-specific in vitro recording of labelled cellsLHb-DRN (top), and schematic showing surgeries site for virus injections to specifically label cellsLHb-DRN (bottom). (B) Schematic showing the optical fiber used for in vitro delivery of blue light (470 nm) and glass electrode used for simultaneous whole-cell recording of labelled cellsLHb-DRN responses (top left). Whole-cell current-clamp recordings from AAV-DIO-ChR2-mCherry-infected cellsLHb-DRN in LHb slices showing 20 Hz_20 ms of blue light stimulation induces tonic firings (middle left). Whole-cell voltage clamp recordings showing long duration (2 s) of blue light stimulation induces temporally precise inward photocurrent (bottom left), Frequency-response curve of membrane excitability to blue light stimulation showing light frequencies ranging from 0.1–20 Hz reliably induce the approximately equivalent firing rate in the ChR2-mCherry expressing cellsLHb-DRN (right). (C) Detailed schematic of the SDS procedure during which the ChR2 expressing cellsLHb-DRN were optically stimulated for 20 min after each exposure to social stress for 7 days, after which the mice underwent the SI test. (D) Cartoon showing in vivo optical stimulation protocol during the SDS paradigm. (E) In the presence of a CD1 social target (nonaggressor), SDS-ChR2 mice display increased time in the corner zone (F2,18 = 3.55, P < 0.05) (right). There was no difference in total travel (right). N = 5–7 mice/group. Error bars: mean ± SEM. The raw data can be found in S11 Data. LHb, lateral habenula; SDS, social defeat stress; SI, social interaction. (TIF) [file pbio.3000709.s005.tif]

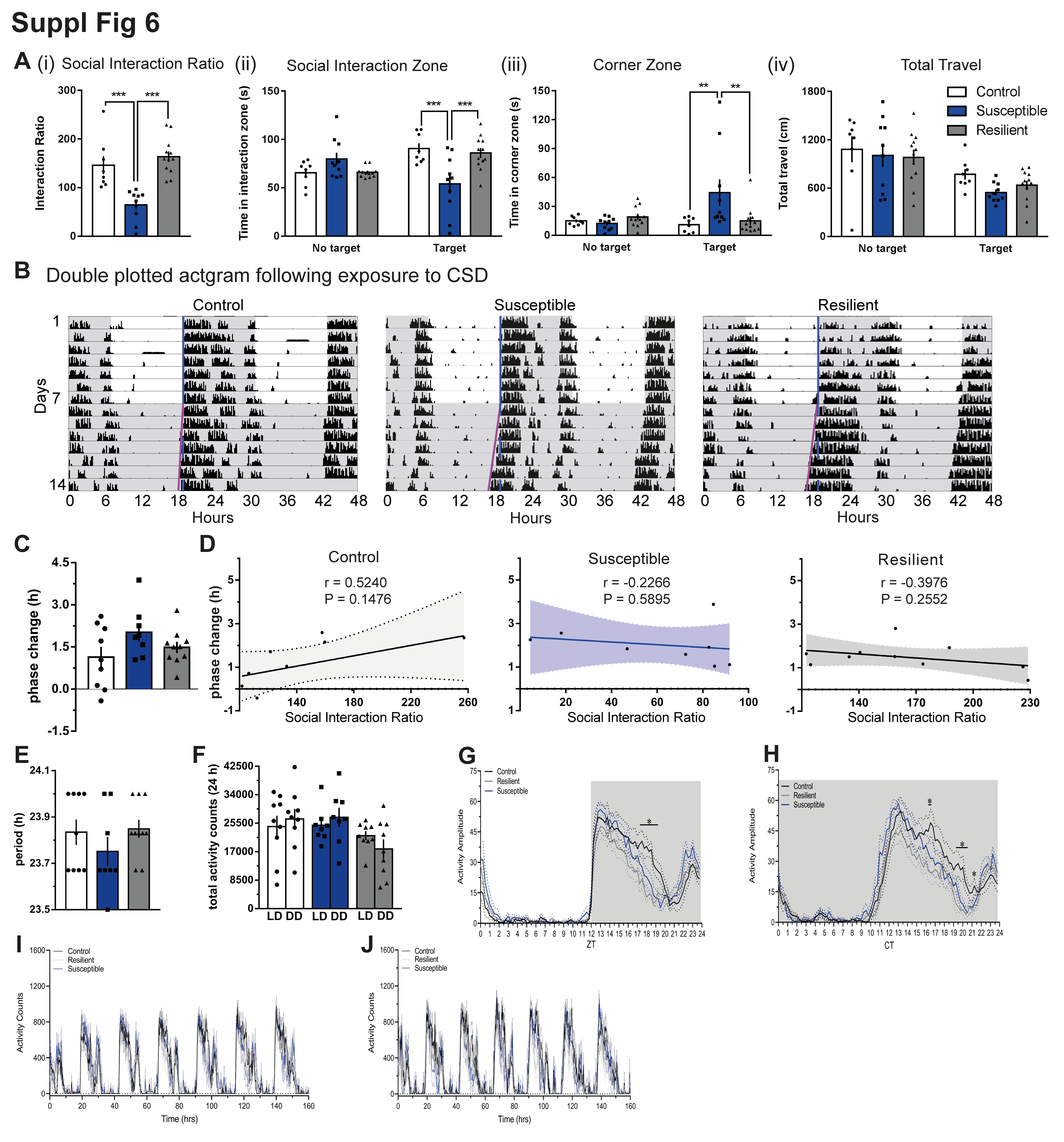

Supplement: S6 Fig — No difference in endogenous rhythms in mice exposed to CSDS. (A) In the presence of a CD1 social target (nonaggressor), susceptible mice displaying (i) decreased SI ratio (F2,27 = 18.08, P < 0.0001), (ii) decreased time in the interaction zone (F2,27 = 19.68, P < 0.0001), (iii) increased time in the corner zone (F2,27 = 5.188, P < 0.05), (iv) no difference in total travel. (B) Representative double-plotted actograms of control, susceptible, and resilient mice. Grey background indicating either dark phase of the LD cycle or DD; light blue and pink lines representing extended regression line derived by onset of activity under LD and DD cycle, respectively. (C–F) No difference in (C) phase change in activity onset in DD, (D) in correlation between interaction ratio and degree of phase change, (E) in free running period length in DD, and (F) in total activity counts in 24 h between LD and DD between control, susceptible, and resilient mice. Though not significant, susceptible mice exhibit slightly larger phase change (C) and shorter free running period (E). (G, H) Intradaily variability was lower in stress-exposed mice compared to stress-naïve groups (P < 0.05). (I, J) There was no difference in interdaily stability between the 3 phenotypes. N = 8–12 mice/group. Error bars: mean ± SEM. The raw data can be found in S12 Data. DD, complete darkness; CSDS, chronic social defeat stress; LD, light–dark; SI, social interaction. (TIF) [file pbio.3000709.s006.tif]

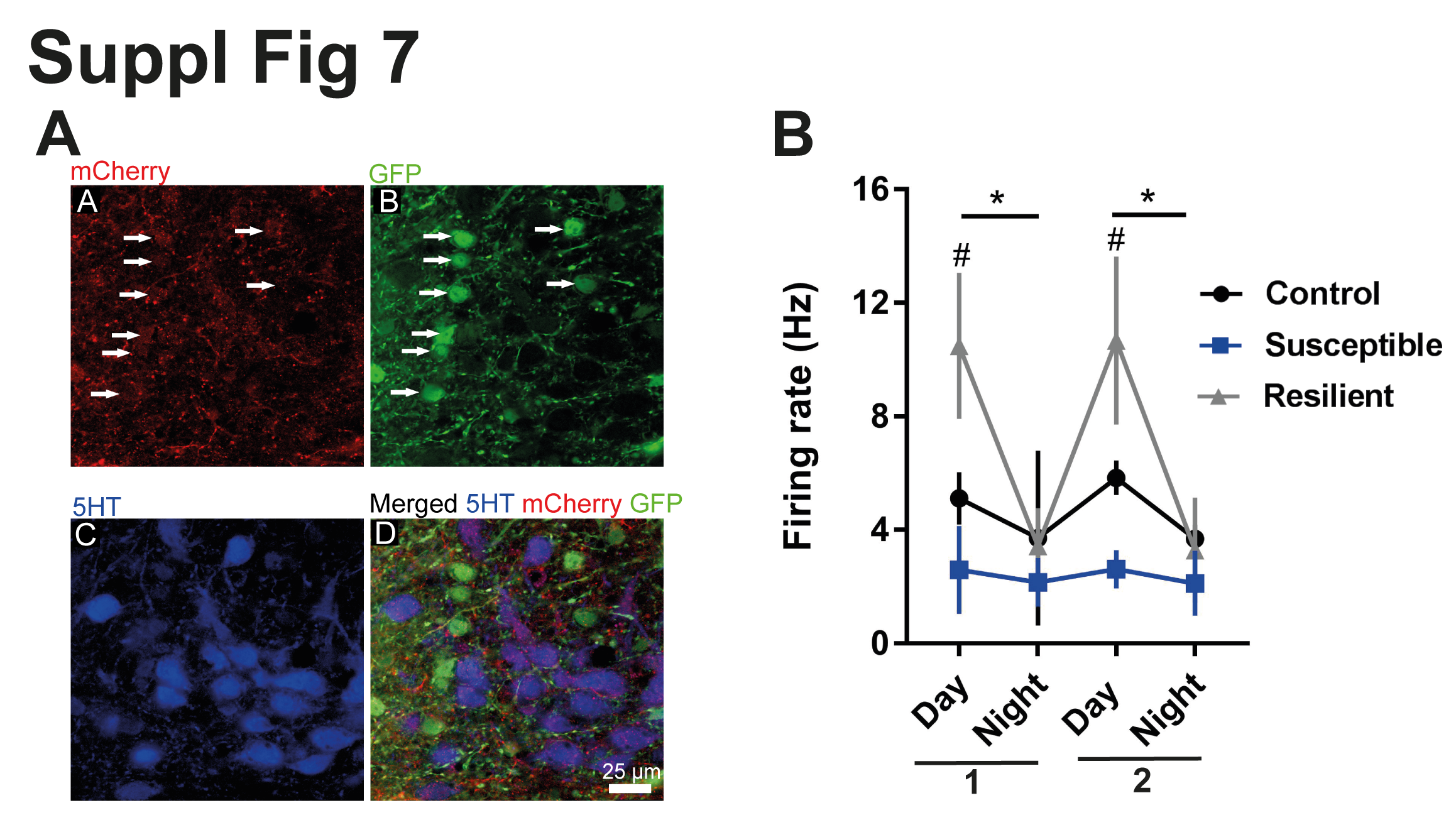

Supplement: S7 Fig — LHb projections innervate both serotonergic and GABAergic neurons in the DRN. (A) Immunohistochemical staining in DRN showing mCherry (red) (A) that represents axons projecting from the LHb infected with AAV2.1-hSyn-hChR2(H134R)-mCherry. The enriched dotted staining on the soma for both GFP-positive GABAergic neurons (B in green as indicated by arrows), and 5HT-positive serotonergic neurons (C in blue). GFP expression was driven by GABAergic-specific mDlx enhancer in DRN infected with AAV2.1-mDlx-GFP. The 3 colours were merged in D. The amount of red dots on the blue serotonergic cells are compatible to those on the green GABAergic cells, suggesting that LHb projections into DRN likely innervate to both DRN GABAergic and DRN 5-HT cells. (B) The in vitro electrophysiology experiments were performed over 16 days after the SI test. To better visualize the rhythmic changes in spontaneous firing in cellsLHb→DRN, data were regraphed where day and night firing was binned into first half (1–8 Day/Night after SI) and the second half (9–16 Day/Night after SI) recording sessions. DRN cells display daily rhythms in spontaneous firings in resilient mice with significantly higher daytime activity than night (Day 1—F2,79 = 4.125, P = 0.0234; Day 2—F2,79 = 4.111, P = 0.0240; n = 53–64 cells from 4 to 10 mice/group). Also, in daytime, spontaneous firing of DRN cells was significantly higher in resilient mice than susceptible (Day 1—F3,79 = 4.085, P = 0.0137; Day 2—F3,79 = 4.072, P = 0.0141; n = 53–64 cells from 4 to 10 mice/group). *day vs night in resilient mice, #day vs day in resilient vs susceptible mice. Error bars: mean ± SEM. The raw data can be found in S13 Data. DRN, dorsal raphe nucleus; GFP, green fluorescent protein; LHb, lateral habenula; SI, social interaction. (TIF) [file pbio.3000709.s007.tif]
