# Supplementary material for: Blunted diurnal firing in lateral habenula projections to dorsal raphe nucleus and delayed photoentrainment in stress-susceptible mice
Source: PLoS Biol. 2021 Mar 10;19(3):e3000709. doi: 10.1371/journal.pbio.3000709 (PMC7984642; doi:10.1371/journal.pbio.3000709)
Supplement: Attachment — Submitted filename: Response_to_Reviewers_PLOS Biology_Resubmission.docx [file pbio.3000709.s021.docx]

PLOS Biology

*****************************************************
REVIEWS:

Reviewer #1 (Mario A. Penzo, signed review): In their manuscript entitled "Blunted Diurnal Firing and Delayed Photoentrainment in Stress-Susceptible Mice" Liu and colleagues use the chronic social defeat stress paradigm (CSDS) to show that susceptibility to stress is associated with changes in the spontaneous firing rates of LHb neurons that project to the DRN. In addition, the authors show that in naïve and resilient mice, LHb-DRN neuron display diurnal variations in firing rates. Importantly, this daily rhythm is blunted in susceptible mice. The authors also explored the potential contribution of specific conductances to diurnal variations in firing, but from the authors' perspective the results from these experiments seem counter-intuitive and somewhat difficult to interpret. However, I believe that some of their results in this category should be explored further, as they could offer insight on the potential mechanisms underlying susceptibility (more on this below). The
authors also show that stress (both susceptibility and resiliency) is associated with changes in entrainment to light advances and that these changes are different in susceptible and resilient subjects. Finally, the authors attempt to draw parallels between susceptibility-associated changes in spontaneous firing rates in the LHb and those observed in the DRN. These later aspects of the study (entrainment and DRN electrophysiology), however, are substantially underdeveloped. As a result, the Discussion section is highly throughout. This leaves the reader with the impression that drawing concrete conclusions from the result presented here is rather difficult. As such, while I find that the study, and in particular its core finding, would be of interest to a wide scientific audience, some critical data is missing here. In particular, the author's need to strengthen the mechanistic aspects of their study, by causally linking cellular and circuit mechanisms to behavior. It is the
reviewer's impression that this is a reasonable expectation that should be met for publication in PLOS Biology. Below is a point-by-point review with critiques and recommendations:

1)      The authors mention that SI scores for control, resilient and susceptible mice were comparable at day and night times. However, it seems that some diurnal variability exists in the naïve group, with animals at night showing higher SI scores. Are the behavior tests (CSDS) and SI also occurring at different ZT times? I am trying to figure out if only the recordings happen at this time or if behavioral manipulations are similarly restricted in a diurnal fashion. If they are, then the behavioral effects observed in the naïve group seems contradictory because LHb neurons have higher firing rates at night. Have the authors run comparisons between these two groups to see if there are statistically significant differences in naïve groups? Given that the N is low for these behavioral tests (since these are subjects used for electrophysiological studies) more data will be needed to achieve more statistical power.

Mice that were used for either day-time or night-time electrophysiology recordings all underwent CSDS between ZT9-10 while the SI tests were performed between ZT 7-9. Daytime electrophysiology recordings were carried out between ZT 2-7 and night-time electrophysiology recordings were carried out between ZT 14-19.

I have added information detailing the times for the CSD, SI and electrophysiological recordings in the relevant sections in the methods.

Though the social interaction (SI) scores for stress naïve (control) mice where electrophysiology recording was conducted in the day (Supplemental Fig. 2A) is visually lower than that of stress naïve mice where electrophysiology recordings were performed in the night (Supplemental Fig. 2B), our t-test statistical comparison did not show any significant difference between the stress naïve groups; t(7)=2.3, p=0.06. Moreover, statistical comparison between susceptible and resilient groups did not show any significant difference within the respective groups: Susceptible- t(8)=0.5, p=0.6 ; Resilient-t(4)=0.1, p=0.9. A possible reason that the stress naïve groups in our study appear to show weak average differences in SI ratio is due to natural variance in SI ratio in stress naïve mice where for example previous published work showed that that stress-naïve mice exhibited broad range of SI ratios. For example, it has been shown that some mice exhibit values as low as 25, while other mice exhibit values as high as 300 (mean value 130) (1). I have also compared SI values between the stress naïve group from the current manuscript (Control – Day / Night Ephys Recordings) together with another behavioural experiment from our lab where we used a much larger sample size because here, we collected brain samples from different ZT’s for genomic analysis (Control – Genomics Experiments). We have added the figure below which we hope will convince the reviewer that the distribution of SI ratio in stress naïve group can be large (Figure A). We also performed one-way ANOVA on all these data sets and we report no significant difference between the stress-naïve mice; F(2,46)=2.27; p=0.11. We have also ensured that the y-axis for the social interaction, corner zone and total travel graphs in supplemental Figure 2 are in the same scale for mice that underwent day or night recordings.

2)      Data in Figure 2 demonstrate that the firing rates of susceptible neurons do not fluctuate as much, as the abstract suggests. But, unlike naïve and resilient mice (in which firing is higher during daytime), firing rate in susceptible seems larger during daytime compared to nighttime. So, diurnal variations in LHb neurons of susceptible mice are not only blunted but inverted. This is an important result, that is only briefly mentioned in the figure legend of Supplementary Figure 2, but mostly ignored throughout the study. What is the reason for this inversion?

We thank the reviewer for highlighting this interesting observation. We performed a 1-way ANOVA to compare spontaneous firing rates between all behavioural phenotypes in the day and the night as shown in figure 2D and E. Statistical analysis showed significant differences in firing between the day and the night; F(5,97)=5.5; p=0.0002. Moreover, post-hoc analysis showed that spontaneous firing in the day for control and resilient mice was significantly lower than spontaneous firing in control and resilient mice in the night (control-day vs control-night: p=0.02; resilient-day vs resilient-night: p=0.02). In contrast post-hoc comparison between susceptible mice in the day and the night showed that spontaneous firing in the day was significantly higher than the night (p=0.008). Thus, the comparison of spontaneous firing does indeed show a significant inversion of diurnal rhythmic activity in susceptible mice compared to control and resilient mice.

We have added the new statistical analysis comparing the relative firing in the day and night between the three phenotypes. Moreover, we discuss mechanisms that may lead to the inversion of diurnal firing in susceptible mice by focusing primarily in putative abnormalities in diurnal clock gene expression and hormone levels.

3)      I was particularly surprised that the authors largely neglected their I-h results. While I-h conductance doesn't show diurnal rhythms in LHb-DRN neurons, stress seems to increase I-h in both susceptible and resilient mice, but the effect is apparently larger in susceptible subjects. Given that I-h is known to contribute to increased excitability, for example in the adjacent paraventricular nucleus of the thalamus (Kolaj et al., 2012; Journal of Neurophysiology), this conductance is a major suspect for the marked increase in daytime firing rates displayed by LHb neurons of susceptible mice. The authors could assess the contribution of this conductance from neurons across all three conditions at both day- and nighttime. This can be achieved by comparing the effect of the I-h inhibitor ZD7288 at both times of the cycle. In addition, the authors could attempt genetic manipulations targeting I-h to investigate the relationship between increased I-h and stress susceptibility. I am not entirely sure why the authors haven't explored this avenue further, given the senior author's previous research on this topic.

We thank the reviewer for this very important point. We did plan to do these experiments for a more detailed paper as a follow up of this manuscript. However, in response to the reviewer’s valid points we investigate the effects of the HCN channel blocker ZD7288 on spontaneous firing in LHb cells projecting to the DRN in control, resilient and susceptible mice in the day and the night. For our studies we investigated the effects of 10μm ZD7288 on spontaneous firing since previous work had shown that this concentration of drug attenuated approximately 50-60% of I*_h_*-currents (2). We did not use higher concentrations as we were worried that if there were indeed small differences in the functional role of HCN channels in driving spontaneous excitability in these cells, very large concentration of ZD7288 may override these subtle differences by completely blocking these channels. We had previously shown that susceptible mice exhibited significantly larger I*_h_*-currents compared to control and resilient mice during the day (Figure 3A). Thus, we predicted that increased daytime excitability in the susceptible mice may be driven, in part, by larger I*_h_*-currents as they shorten the rebound spike latency leading to a more rapid return to the resting state such that the cell is ready to fire again. We report that addition of ZD7288 attenuates spontaneous firing in some cells but in others it increased spontaneous firing. We found that the percentages of excited and inhibited cells in control, susceptible and resilient mice during the day were: 63%:37%, 50%:50% and 55%:45% respectively. For nigh-time recordings the percentages of excited and inhibited cells in control, susceptible and resilient mice were 50%:50%, 50%:50% and 67%:33% respectively (Supplemental Figure 4A). The bidirectional effect of blocking HCN channels on spontaneous firing has been reported previously in different cells (3-5). For example, one study reported that approximately 50% of globus pallidus (GP) cells increased firing following ZD7288 administration (5).

HCN channels increase spontaneous firing by shortening the amplitude and duration of the afterhyperpolarization (AHP) which then enables a cell to return to the resting state quicker and thus shorten the interval between action potentials (6). Therefore, the mechanism by which ZD7288 decreased spontaneous firing in the subset of LHb-DRN cells is likely via the blockade of somatic HCN channels leading to increased amplitude and duration of the AHP which prolongs the time it takes a cell to return to its resting state in order to fire again. How ZD7288 induced block of HCN channels leads to increased firing is more complex. For example, in reticular thalamic neurons ZD7288 induced block of HCN2 channels was shown to enhance spontaneous firing as a result of the interaction between HCN2 channels and AMPA receptors at the dendritic tree (7). This study showed that dendritic HCN2 channels are co-localized with AMPA receptors and that activation of these channel increases local membrane conductance that leads to decreased local membrane time constant which increases the rate of decay of excitatory AMPA-receptor induced post-synaptic potential (EPSP). In contrast ZD7288 induced block of HCN2 channels increases the local dendritic membrane time constant that delays the decay of rate of AMPA receptor induced EPSP. This increases the temporal summation of EPSP that progressively depolarises the membrane potential resulting in increased the probability of firing. Thus, it is possible that in those cells that bath application of ZD7288 induced increased spontaneous firing likely express higher percentage of dendritic HCN2 channels that is co-localized to AMPA receptors. In contrast those cells that decreased spontaneous firing following bath application of ZD7288 may express higher percentage of somatic HCN channels.

We did not observe significant differences in the inhibitory or excitatory effects of ZD7288 between the 3 phenotypes in the day or the night (Supplemental Figure 4B-C). This would suggest that I*_h_*-currents most likely do not play a large role in driving the increased excitability in susceptible mice during the day. It is most likely that the increased day-time spontaneous firing observed in susceptible mice is driven by other ion channels. A possible explanation for increased firing in susceptible mice could be due to elevated expression of subtypes of potassium channels that are known to increase spontaneous firing by shortening the duration of an action potential (see below).

We also investigated the potential role of voltage sensitive potassium (K_V_^+^) channels in driving the increased day-time spontaneous firing in LHb-DRN cells of susceptible mice. Though most Kv^+^-channels tend to decrease firing in cells, there are subsets of Kv^+^-channels such as the A-type (IKA), delayed rectifier (DR) and large conductance voltage and calcium activated (BK) channels that can increase cell firing (8-10). For example, previous work had shown that delayed rectifier currents (DR) drive diurnal excitability in SCN cells (8). We had previously shown that peak and sustained K^+^-currents were larger in the day in susceptible mice (Figure 3C-D) and this led us to speculate that fast kinetic IA and slower kinetic DR and BK K^+^-currents may be driving increased firing in susceptible mice. Thus, we investigated the effect of DR and BK K^+^-channel blocker tetraethylammonium (TEA: 1mM) on spontaneous firing in cells^LHb→DRN^ from control, susceptible and resilient mice in the day and the night. We showed that TEA attenuated firing in a subset of cells while in others it increased firing (Supplemental Figure 4D). TEA significantly attenuated spontaneous firing in susceptible and resilient mice compared to stress-naïve control mice during the day (Supplemental Figure 4E). TEA induced attenuation of spontaneous firing in susceptible mice may be indicative of increased inhibition of putative DR and Bk channels, both of which are blocked by TEA. The increased inhibition in resilient mice is less clear as we did not observe large Kv^+^-currents in these mice during the day. A possible explanation may be that TEA is blocking other channels that drive excitability (11). We are planning future experiments to further investigate this. Though not statistically significant, at night we observed that TEA-induced attenuation of firing was larger in control mice compared to susceptible and resilient groups. Moreover, the degree of inhibition in night control mice was significantly larger than in day control mice. This was unexpected as we did not show diurnal variations in Kv^+^-currents in control mice. It is possible that at night there is a larger expression of BK channels that is blocked by TEA. As these channels are also activated by Ca^2+^, it is possible that the depolarizations steps alone in our previous electrophysiological recordings did not sufficiently activate night time BK channels. Though not statistically significant, % inhibition in susceptible-mice was larger in the day compared to the night while the average % inhibition in resilient mice was the same in the day and night. This correlates with our previous observation of Kv^+^-current recordings. Since TEA can also block a variety of K_V_^+^ channels, that functionally decrease cell firing, it was not surprising that addition of TEA increased spontaneous firing in a subset of cells (Supplemental Figure 4F). In both day and night, the % excitation was significantly higher in susceptible mice compared to control and resilient groups which is likely indicative of larger expression of K_V_^+^-channels that typically supress spontaneous firing. Our previous electrophysiological recordings showed similar K_V_^+^-current in all phenotypes at night. However, our observation that susceptible mice exhibit greater % excitatory effect following application of TEA was unexpected. One possible explanation may be that removel of inhibitory tone increases excitatory tone to a greater degree in these cells. We are planning extensive detailed future studies using more specific blockers to examine the potential role of the subtypes of Kv^+^-channels in driving diurnal excitability in cells^LHb→DRN^ in stress exposed mice.

We have added relevant description of the results and figures in the results section and added a section in the discussion to discuss the main findings following addition of ZD7288 and TEA.

4)      As mentioned above, a major limitation of this work is the lack of manipulations to link individual findings. This makes the paper feel as composed of attractive vignettes that are not linked into a cohesive story. For example, data presented in Figure 5 shows that stress affects circadian rhythms but that susceptible and resilient mice show somewhat opposing phenotypes. So, while susceptible mice show initial delay in entrainment but ultimately entrain in a similar timeframe as controls, resilient mice seem to entrain significantly faster than controls. This result is interesting when treated as an independent observation. However, there is no evidence linking LHb activity to these different phenotypes. As such, it is unclear that the stress induced firing phenotypes seen in LHb and explored in Figures 1 and 2 are related to the circadian phenotype.

We agree that our data does not directly correlate changes in cells^LHb→DRN^ firing to changes in rate of photoentrainment. However, though the evidence is not conclusive, we feel that this initial piece of evidence that susceptible mice exhibit elevated firing in cells^LHb→DRN^ and show slow initial rate of photo-entrainment is worth presenting as it opens up future investigations into potentially novel circuits that regulate mood and daily rhythms. This will help develop a better understanding of the bi-directional association between mood disorders and changes in daily rhythms. Because of time and current technical constraints, we have not been able to perform the key experiments needed to show the direct association between abnormal firing in cells^LHb→DRN^ and changes in photo-entrainment to a new Light cycle. We are planning new experiments for a follow-up manuscript to further investigate whether changes in circuits including cells^LHb→DRN^ in susceptible mice is directly related changes in the rate of photoentrainment. For these studies we will use c-fos, in-vivo imaging, chemogenetic and optogenetic approaches.

5)      A similar issue arises when evaluating the DRN data. While this data is very interesting on its own, the parallels drawn between decreased spontaneous firing in the DRN and increases firing in the LHb in susceptible mice is only speculative. Indeed, the authors suggest that feedforward inhibition of DRN neurons resulting from increased LHb-DRN activity is the most parsimonious interpretation of the susceptible state. But evidence that this is the case is missing. Given the authors' technical expertise I am confident that this is something they can address experimentally. Why leave it to speculation?

The reviewer raises an important point. We had planned on performing opto-electrophysiology experiments to determine functional circuit connections between LHb terminal in the DRN and putative DRN GABAergic interneurons and DRN 5-HT neurons. We injected AAV2.1-hSyn-ChR2-(H134R)-mCherry into the LHb to label neuronal LHb cells and AAV2.1-mDlx-GFP, provided by Gord Fishell’s lab, into the DRN to label DRN GABAergic interneurons (dlx promoter which results in specific expression in DRN GABAergic interneurons). We had then planned to make DRN slices for in-vitro optoelectrophysiology recordings. Specifically, we would have performed whole cell path-clamp recordings in labelled putative DRN GABAergic cells and un-labelled putative DRN 5-HT cells following optical stimulation (blue light) of LHb terminals in the DRN. We predict that LHb terminals makes direct and in-direct (via GABAergic interneurons) contact with putative 5-HT cells. To test this prediction, we would have recorded from: (i) putative labelled GABAergic interneurons and (ii) putative 5HT cells. LHB cells are predominantly excitatory. Thus, if LHb terminals innervate DRN GABAergic cells we would have likely recorded EPSP’s in DRN GABAergic cell. Moreover, indirect innervation of LHB to 5-HT cells (via GABAergic cells) would lead to IPSP’s in 5-HT cells (with a longer time delay of post-synaptic current onset) while direct innervation of LHb to 5-HT cells would lead to EPSP’s in 5-HT cells (with a shorter onset of post-synaptic current). Unfortunately, we experienced 2 technical issues: (i) viral expression of ChR2-mCherry in LHb terminals in the DRN was not robust enough for us to stimulate with blue-light in order to activate LHb terminals. This was likely due to mis-targeting and/or bad viral batc and, (ii) Low GFP expression in GABAergic cells. This is because GFP expression for this vector starts to disappear 2 weeks post infection. Because of pandemic related lab delays we waited 5 weeks prior to starting the opto-electrophysiology experiments at which point GFP labelling was extremely low. We are planning to repeat this experiment for follow up studies in the near future.

We had also performed some preliminary immunohistochemical experiments to investigate whether LHb terminals are localized close to putative DRN GABAergic interneurons or putative DRN 5HT neurons. AAV2.1-mDlx-GFP provided by Gord Fishell’s lab was injected into the DRN. This vector is under control of the dlx promoter which results in specific expression in DRN GABAergic interneurons. AAV2.1-hSyn-(H134R)-mCherry was injected into the LHb in order to express mCherry in all LHb neuronal cells. Immunohistochemical labelling in the DRN visualized LHb axons/terminals (mCherry), GABAergic interneurons (GFP) and putative 5HT cells (Blue). Our preliminary immunohistochemical data shows that LHb terminals surround both putative GABAergic interneurons and 5HT projections cells in the DRN (Supplemental Figure 7A). This implies that glutamatergic LHb terminals may excite putative 5HT projections cells via direct synaptic contact or inhibit 5HT cells via feed-forward inhibition involving GABAergic interneurons. An intriguing possibility worth examining would be to determine whether susceptible and resilient mice have differences in weight of direct or indirect contact between the LHb and DRN 5HT cells. We have added this into the discussion. Moreover, we are planning more detailed anatomical analysis together with functional micro-circuit analysis using opto-electrophysiology as previously mentioned.

6)      Related to the previous point, are the DRN effects exclusive to 5-HT neurons? The authors should consider selectively assessing these effects while contrasting GABA and 5-HT DRN neurons. Overall, I believe that addressing these issues will allow the authors to achieve a more rounded study that doesn't seem so speculative. One possibility (if other reviewers agree) would be to remove the entrainment data and focus on linking the LHb and DRN phenotypes. In addition, as stated above, the authors must address the possibility that some of the identified conductances, in particular I-h, play a fundamental role on stress susceptibility.

The reviewer raises another interesting and relevant point. During the course of this study we started considering the functional role of LHb input to DRN 5HT and non-5HT cells since previous studies have already shown that DRN cells have subpopulations of non-5HT cells. We were unable to start any preliminary analysis for the current paper. We are planning these interesting studies for a follow up paper.

Minor:
1)      Consider combining Figure 1 and Supplementary Figure 1.

We have added the cartoon of the CSD and SI paradigm and social interaction ratio data in Figure 1. We feel that the rest of Supplemental Figure 1 should remain as it is.

2)      Also, consider citing the Kolaj et al., 2012 (Leo Renaud's lab) study about diurnal variations in spontaneous firing in the neighboring paraventricular thalamus. That study found that, unlike in the LHb (present stud), diurnal changes in I-h occur in naïve mice.

We have cited Kolaj et al., 2012 in our discussion as it is relevant to our paper.


Reviewer #2: In this study, Liu et al. report that the excitability of DRN-projecting LHb cells in stress-susceptible mice is significantly increased during the day, while the excitability of DRN cells is significantly reduced. They also find that specific activation of the DRN-projecting LHb cells during the day induces stress-susceptibility. Finally, they report that stress induces blunted daily rhythms in the firing of DRN-projecting LHb cells. Although the experiments appear to be well-controlled and interpretable, there are large number of technical and conceptual issues that suggest that many of the present experiments are not up to the standards of this journal.

Major points:
1.      Previous studies found that exposure to stress significantly increased burst firing in LHb cells (Yang et al., 2018; Huang et al., 2019). In contrast, the authors found that exposure to CSDS significantly decreased burst firing in LHb cells (Fig. 2H). The authors should explain this apparent discrepancy.

We thank the reviewer for this valid point. Both Yang et al., 2018 and Huang et al., 2019 showed that burst firing was increased in random LHb cells in mice exposed to chronic restrained stress while in our study we recorded electrophysiological characteristics specifically in LHb cells projecting to DRN (cells^LHb-DRN^) in mice exposed to chronic social defeat stress (10mins social stress / 15 days). Though we still show increased firing (in day) in stress-susceptible mice compared to resilient and control mice our observation that burst activity was lower in susceptible compared to resilient and control groups is indeed unexpected. Reasons for this could be:

(1) We recorded activity specifically in cells^LHb→DRN^ while both Yang et al., 2018 and Huang et al., 2019 recorded in random LHb cells irrespective of their projections. Previous work had shown differences in ion channel expression in projection specific cells where for example VTA cells projecting to NAc express I*h*-currents while VTA cells projecting to the mPFC did not (12, 13). Moreover, we had previously shown projections specific differences in firing properties in susceptible mice where for example VTA cells projecting to NAc showed increased firing while VTA cells projecting to the mPFC showed decreased firing (14). Thus, it is likely that different LHb projection cells can express different ion channels and so potentially undergo different physiological responses to stress exposure. Though we don’t yet have the evidence for this, it is possible that in cells^LHb→DRN^ of susceptible mice there is increased expression of channels that attenuate burst firing while the increased burst firing reported by Yang et al., 2018 and Huang et al., 2019 may be indicative of activity in a mixed population of LHb cells projecting to numerous target sites which may give an average increase in burst firing.
(2) Previous work had shown that different stressors can have differing effects on activity in cells, where for example weaker stressors had decreased activity of mesolimbic cells while stronger stressors increased activity (15). Moreover, we had previously shown that exposure to chronic social defeat stress (CSDS) induced increased activity in mesolimbic cells in susceptible mice (14) while chronic mild stress induced decreased activity in mesolimbic cells (16). Thus, it is possible that the decreased bursting in susceptible mice that we report may be due to physiological responses to the CSDS paradigm while increased bursting observed by Yang et al., 2018 and Huang et al., 2019 may be due to differential physiological responses to the restrained stress paradigm used by these groups.
(3) Yang et al., 2018 and Huang et al., 2019 presented data for all mice exposed restrained stress while in our study we separated stress exposed mice to susceptible and resilient phenotypes. Interestingly at night we show increased bursting in resilient mice compared to control and susceptible groups. There may be a diurnal component that promotes greater bursting-like responses to stress at night specifically in cells^LHb→DRN^. We plan to investigate this in future studies.

2.      The authors reported that susceptible mice showed altered Ih-current and Kv+ currents in DRN-projecting LHb cells, accompanied by elevated diurnal, spontaneous firing. However, the causal relationships between the changes of Ih-current/Kv+ currents and the spontaneous firing of LHb cells are still lacking.

The reviewer has raised a very good point. We have performed some pharmacological experiments in an attempt to correlate firing with altered I*_h_*-currents and Kv^+^-currents. We have given a more detailed response to reviewer 1 question 3 as they had asked the same question. We have also added additional information in the results and discussion section.

3.      The authors found that specific activation of DRN-projecting LHb cells induced stress-susceptibility. It is possible that DRN-projecting LHb cells may also project to mood-related brain regions other than the DRN. To further confirm whether the LHb-DRN pathway is implicated in stress-susceptibility, the authors should regulate neuronal activity of DRN that can receive direct input from the LHb and exam its effects on depressive-like behaviors.

The author raises a very important point.

(a) For ephys recordings of control, resilient and susceptible mice in day and night we first injected conditional DIO-EYFP into the LHb and retrograding AAV-Cre into the DRN. The conditional EYFP virus will only express when Cre is present. Since the AAV-Cre injected into the DRN is retrograding, it will be taken up by all inputs into the DRN. However, since DIO-EYFP is only present in the LHb, only those LHb cells projecting to DRN will express EYFP. We only recorded in EYFP expressing cells and are thus are confident that all our recordings are of LHb cells projecting to the DRN.
(b) For optogenetic recordings we injected DIO-ChR2-EYFP into the LHb and retrograding AAV-Cre into the DRN. The conditional ChR2-EYFP virus will only express when Cre is present. Since AAV-Cre injected into the DRN is retrograding, it will be taken up by all inputs to DRN. However, since DIO-ChR2-EYFP is only present in the LHb, only LHb cells projecting to DRN will express ChR2-EYFP. Thus, we are confident that optical stimulation in the LHb specifically stimulated LHb-cells projecting to the DRN. We and others have used this approach extensively and are confident with the reliability of projection specific expression (14, 17-19).

For both point (a) and (b) we carried out extensive surgical validations prior to starting the studies to confirm the accuracy of our coordinates used for viral surgery. For this we used a combination of India Ink to initially validate targeting of LHb and DRN nuclei. This was followed by injecting retrograding luma beads into the DRN to confirm LHb labelling. Moreover, we validated surgical targeting for: (a) by checking the injection site in the DRN and LHb after we prepared slices for electrophysiological recordings and (b) by checking the injection site in the DRN and LHb upon completion of the behavioural assay.

Though we agree that there is always some element for errors and uncertainty we are confident that the majority of our surgeries reliably labelled LHb cells projecting to the DRN.

The reviewers interesting question also bring up another interesting point. Do LHb cells that project to DRN also project to a secondary site? If so, then indeed our optical stimulation experiment would lead to activation of other downstream targets. We will investigate this by performing the same surgeries as described in a and b followed by slicing other brain regions to determine whether we see EYFP terminals coming from the LHb. We will plan this in the near future.

4.      The authors found that the spontaneous firing of DRN-projecting LHb cells was significantly increased, while the spontaneous firing of DRN cells was significantly decreased. It is well documented that most of LHb cells are excitatory. Why the enhanced excitatory inputs to the DRN can reduce the spontaneous firing of DRN cells? What is the identity of the recorded DRN? Are they directly innervated by the LHb?

As mentioned in response to reviewer 1 Question 5 we had attempted to perform opto-electrophysiology experiments to investigate synaptic connections between LHb terminals in the DRN and GABAergic cell and putative 5HT cells in the DRN. Due to technical issues we were unable to perform these expereimnts. We are planning to repeat these experiments which we will incorporate in our next manuscript.

We did perform some preliminary immunohistochemical analysis that shows LHb terminals localized close to GABAergic and putative 5HT cells in the DRN. A more detailed description is provided in our response to reviewer 1 Question 5. Moreover, we have added the information to our manuscript.

5.      The authors performed in vivo recordings in anesthetized mice. Anesthesia may affect firing of neurons (Schonewille et al., 2006). The authors should conduct the in vivo recording experiments in behaving animals.

We appreciate the valid point raised by the reviewer and understand that recordings in awake behaving mice would be the best indicator of neural firing in the natural state. However, the purpose of the in-vivo recording we performed was simply to determine whether susceptible and resilient mice exhibited differences in basic spontaneous firing in LHB cells in an intact brain. Moreover, this approach has been successfully used previously in the mesolimbic circuit as an initial markers of stress induced changes in brain circuits before slice physiology experiments were then performed (18, 20). Though spike characteristics are indeed affected by anaesthesia as described by Schonewille et al., 2006, they report that the overall simple spontaneous firing rate in Purkije cells was not different between awake and anesthetized rats as shown in Fig 1d and 2b of their manuscript. Thus, we feel for the current study our preliminary in-vivo approach was valid for determining the effects of social stress on basic firing rate in the LHb.

We have recently purchased the fiber-photometry system and are indeed planning to investigate differences in LHb-DRN circuits in control, resilient and susceptible mice in awake behaving mice. These studies will give us a more accurate measurement of diurnal changes in the circuit in behaving mice. This will be for a future detailed study.

Minor point:
1.   The authors should explain why they choice tonic stimulation instead of phasic stimulation in the optogenetic stimulation experiment.

We choose tonic stimulation because our slice data showed that susceptible mice exhibited increased tonic firing in the day, while bursting firing was in fact decreased. Moreover, we did not show any differences in burst firing during our in-vivo recordings between the control, resilient and susceptible mice. We had found from our slice recordings that susceptible mice exhibited tonic firing with frequency of around 20Hz. Thus, for the optogenetic study we first piloted a small experiment to investigate the effect of 20Hz tonic stimulation and found that this protocol induced the susceptible phenotype. We then proceeded with a larger experiment using this protocol.

2.   There are a few typos throughout the manuscript. The authors should carefully proof the manuscript.

We apologies for our typos. We have gone through our manuscript carefully and correct our mistakes.

References:
1. Yang Y, et al. Ketamine blocks bursting in the lateral habenula to rapidly relieve depression. Nature. 2018 Feb 14;554(7692):317-322.

2. Huang L, et al. A Visual Circuit Related to Habenula Underlies the Antidepressive Effects of Light Therapy. Neuron. 2019 Apr 3;102(1):128-142.e8.

3. Schonewille M, et al. Purkinje cells in awake behaving animals operate at the upstate membrane potential. Nat Neurosci. 2006 Apr;9(4):459-61;


Reviewer #3: This paper describes a detailed and very elegant series of experiments exploring the role of dorsal raphe nuclei (DRN) projecting neurons of the lateral habenula (LHb) in a chronic social defeat stress (CSDS) mouse model of depression. The key findings are that stress-susceptible mice show a blunting of diurnal firing patterns in the LHb>DRN pathway, and that even sub-threshold stressed animals can be made to show depressed behaviour via stimulation of this circuit. Furthermore, the authors show that disruption of this circuit in this mouse model of depression is associated with altered circadian photoentrainment. These data provide important insight into the links between the circadian system and specific neuronal circuits in mood disorders.

Overall, the paper is very clearly presented and progresses logically. The authors first characterise LHb firing, then examine the specific Lhb>DRN projections, before characterising the role of specific channels. Via the use of detailed electrophysiological recordings, the authors identify a role for HCN channels and sustained Kv+ channels in response to CSDS. They then show use optogenetic studies on DRN projecting LHb neurons in subthreshold SDS (SSDS) mice, showing that stimulation of these cells is sufficient to produce stress-susceptibility. They then show that whilst circadian rhythms appear intact, that resilient mice show faster re-entrainment to a jet lag style shift in the LD cycle. Finally, they show rhythms in DRN spontaneous firing are also blunted in stress susceptible mice.

The links between the habenula, mood and photoentrainment are currently highly topical given high profile studies on the role of light in regulating mood and the role of the habenula (Fernandez et al., 2018 Cell; Huang et al., 2019 Neuron). This study provides critical insight into the links between mood disorders and circadian disruption, and the characterisation of the LHb/DRN/SCN circuit will be of interest to many research fields.

Specific comments are provided below:

1.      In several places in the paper, changes in spontaneous neuronal activity are compared between control, stress susceptible and stress resilient animals during the day or night. For example, in Figure 2D-E, Lhb>DRN neurons show an increase in daytime firing in stress susceptible mice, but no differences occur during the night. I was surprised that day vs night are not explicitly compared, as this could be informative. For example, in the data in Figure 2D-E, it looks like the normal (control) mice show low firing during the day and higher on a night. A similar pattern is observed in the stress resilient mice. By comparison, the stress-susceptible mice show high firing during the day and lower firing on a night. This suggests that the rhythms in these cells are not just blunted, but actually inverted. I appreciate that there may be technical reasons why direct comparison between electrophysiological data may not be directly compared between preparations, but this may be an
interesting observation.

We thank the reviewer for an extremely pertinent point. We should have compared the differences between the day and the night in the initial submission. We have now performed this analysis and given the details above in response to a similar question raised by reviewer 1 question 5.

We have also added the statistical details for the day/night comparison in the new manuscript and have also discussed possible reasons for the inversion of daily rhythms.

2.      Whilst stress-resilient mice show faster re-entrainment, are stress-susceptible mice actually slower at re-entraining? These seem comparable with controls. Could this faster re-entrainment be as a result of differences in non-photic entrainment in stress resilient mice?

The susceptible mice do re-entrain with a similar rate to control mice from day 3 onwards. However, we consistently see that in the first 2 days of photoentrainment to the new light-dark cycle, susceptible mice are significantly slower at re-entrainig relative to control and resilient mice. We mentioned in the discussion that a possible mechanism for this initial slow photoentrainment in susceptible mice may be due to aberrant SIK1 signalling as this has been shown to regulate photoentrainment (21). We also suggest that possible aberrations in direct or indirect projections from DRN to the SCN may initiate the slow initial rate of photoentrainment. This idea comes from previous reports that show VTA dopamine signalling (another region that regulates mood) to the SCN regulates the speed of photoentrainment (22). This is currently speculative. We are planning experiments to investigate his phenomenon in detail.

As the reviewer suggest, indeed it is possible that differences in the rate of photoentrainment may also be due to non-photic entrainment. It is well established that the DRN plays a role in non-photic entrainment (23, 24). For example, non-photic cues such as temperature arousal and sleep-wake patterns have been shown to be affected in susceptible mice (25-27). Moreover, the DRN has a functional role in regulating body temperature, sleep-wake cycle, arousal (28-30). Thus, it is possible that pathophysiological firing in cells^LHb→DRN^ and DRN cells in general may affect biological processes that regulate temperature, arousal and sleep-wake cycle that also effect photoentrainment in susceptible mice. We have added this very valid point into our discussion.

3.      It may be helpful to the reader to provide a figure showing the proposed interactions in the LHb, DRN and SCN circuit, and how these are affected in depression.

We appreciate the suggestion from the reviewer. However, since: (i) we are in the very early stages of understanding how stress effects diurnal rhythms and (ii) there are still large gaps in our knowledge of the functional connections between mood and biological rhythms we do not feel confident at present in developing a working model of possible interactions between the LHb, DRN and SCN. We are working on developing a model for a future manuscript.

Reviewer #4: The study by Liu et al investigates the role of the lateral habenula neurons that project to dorsal raphe in the impact of stress on circadian rhythms. The authors show that LHB-DRN cells display blunted diurnal firing in mice susceptible to social defeat stress compared to control or resilient conditions. They further show that enhancing activity in LHB-DRN cells using optogenetics during social defeat stress induces social avoidance. Finally, the authors demonstrate that susceptible mice display slower photoentrainment to a shift in LD cycle while resilient mice display a faster photoentrainment. Overall this is an important and rigorous study that provides new information into the circuitry involved in stress effects on circadian rhythms. I only have one minor suggestion below that would help to improve the clarity of the study.


This reviewer understands that SDS protocols are modified across labs based on what is optimal in each laboratory. However, the 7 day SDS would not typically be considered a subthreshold SDS. Perhaps the authors could simply modify their nomenclature to 15D-SDS and 7D-SDS and explain why the 7 days was advantageous for the optogenetic susceptible inducing studies.

We thank the reviewer for the comment and we agree that 7 day of social stress may not be a subthreshold stress. Thus, we have now identified the two forms of stress in the manuscript accordingly:

15 days of chronic social stress (CSDS)

7 days of social stress (SDS).

**Reference**

1.	Krishnan V, Han MH, Graham DL, Berton O, Renthal W, Russo SJ, et al. Molecular adaptations underlying susceptibility and resistance to social defeat in brain reward regions. Cell. 2007;131(2):391-404.

2.	Wu X, Liao L, Liu X, Luo F, Yang T, Li C. Is ZD7288 a selective blocker of hyperpolarization-activated cyclic nucleotide-gated channel currents? Channels. 2012;6(6):438-42.

3.	McDaid J, McElvain MA, Brodie MS. Ethanol effects on dopaminergic ventral tegmental area neurons during block of Ih: involvement of barium-sensitive potassium currents. J Neurophysiol. 2008;100(3):1202-10.

4.	Hawkins VE, Hawryluk JM, Takakura AC, Tzingounis AV, Moreira TS, Mulkey DK. HCN channels contribute to serotonergic modulation of ventral surface chemosensitive neurons and respiratory activity. J Neurophysiol. 2015;113(4):1195-205.

5.	Chen L, Xu R, Sun FJ, Xue Y, Hao XM, Liu HX, et al. Hyperpolarization-activated cyclic nucleotide-gated (HCN) channels regulate firing of globus pallidus neurons in vivo. Mol Cell Neurosci. 2015;68:46-55.

6.	Robinson RB, Siegelbaum SA. Hyperpolarization-activated cation currents: from molecules to physiological function. Annual review of physiology. 2003;65:453-80.

7.	Ying SW, Jia F, Abbas SY, Hofmann F, Ludwig A, Goldstein PA. Dendritic HCN2 channels constrain glutamate-driven excitability in reticular thalamic neurons. J Neurosci. 2007;27(32):8719-32.

8.	Itri JN, Michel S, Vansteensel MJ, Meijer JH, Colwell CS. Fast delayed rectifier potassium current is required for circadian neural activity. Nat Neurosci. 2005;8(5):650-6.

9.	Gu N, Vervaeke K, Storm JF. BK potassium channels facilitate high-frequency firing and cause early spike frequency adaptation in rat CA1 hippocampal pyramidal cells. J Physiol. 2007;580(Pt.3):859-82.

10.	Itri JN, Vosko AM, Schroeder A, Dragich JM, Michel S, Colwell CS. Circadian regulation of a-type potassium currents in the suprachiasmatic nucleus. J Neurophysiol. 2010;103(2):632-40.

11.	Adams CM, Price MP, Snyder PM, Welsh MJ. Tetraethylammonium block of the BNC1 channel. Biophys J. 1999;76(3):1377-83.

12.	Lammel S, Hetzel A, Hackel O, Jones I, Liss B, Roeper J. Unique properties of mesoprefrontal neurons within a dual mesocorticolimbic dopamine system. Neuron. 2008;57(5):760-73.

13.	Lammel S, Ion DI, Roeper J, Malenka RC. Projection-specific modulation of dopamine neuron synapses by aversive and rewarding stimuli. Neuron. 2011;70(5):855-62.

14.	Chaudhury D, Walsh JJ, Friedman AK, Juarez B, Ku SM, Koo JW, et al. Rapid regulation of depression-related behaviours by control of midbrain dopamine neurons. Nature. 2013;493(7433):532-6.

15.	Valenti O, Gill KM, Grace AA. Different stressors produce excitation or inhibition of mesolimbic dopamine neuron activity: response alteration by stress pre-exposure. Eur J Neurosci. 2012;35(8):1312-21.

16.	Tye KM, Mirzabekov JJ, Warden MR, Ferenczi EA, Tsai HC, Finkelstein J, et al. Dopamine neurons modulate neural encoding and expression of depression-related behaviour. Nature. 2013;493(7433):537-41.

17.	Friedman AK, Walsh JJ, Juarez B, Ku SM, Chaudhury D, Wang J, et al. Enhancing depression mechanisms in midbrain dopamine neurons achieves homeostatic resilience. Science. 2014;344(6181):313-9.

18.	Zhang H, Chaudhury D, Nectow AR, Friedman AK, Zhang S, Juarez B, et al. alpha1- and beta3-Adrenergic Receptor-Mediated Mesolimbic Homeostatic Plasticity Confers Resilience to Social Stress in Susceptible Mice. Biol Psychiatry. 2019;85(3):226-36.

19.	Cerniauskas I, Winterer J, de Jong JW, Lukacsovich D, Yang H, Khan F, et al. Chronic Stress Induces Activity, Synaptic, and Transcriptional Remodeling of the Lateral Habenula Associated with Deficits in Motivated Behaviors. Neuron. 2019;104(5):899-915 e8.

20.	Cao JL, Covington HE, 3rd, Friedman AK, Wilkinson MB, Walsh JJ, Cooper DC, et al. Mesolimbic dopamine neurons in the brain reward circuit mediate susceptibility to social defeat and antidepressant action. J Neurosci. 2010;30(49):16453-8.

21.	Jagannath A, Butler R, Godinho SIH, Couch Y, Brown LA, Vasudevan SR, et al. The CRTC1-SIK1 pathway regulates entrainment of the circadian clock. Cell. 2013;154(5):1100-11.

22.	Grippo RM, Purohit AM, Zhang Q, Zweifel LS, Guler AD. Direct Midbrain Dopamine Input to the Suprachiasmatic Nucleus Accelerates Circadian Entrainment. Curr Biol. 2017;27(16):2465-75 e3.

23.	Edgar DM, Reid MS, Dement WC. Serotonergic afferents mediate activity-dependent entrainment of the mouse circadian clock. Am J Physiol. 1997;273(1 Pt 2):R265-9.

24.	Marchant EG, Watson NV, Mistlberger RE. Both neuropeptide Y and serotonin are necessary for entrainment of circadian rhythms in mice by daily treadmill running schedules. J Neurosci. 1997;17(20):7974-87.

25.	Olini N, Rothfuchs I, Azzinnari D, Pryce CR, Kurth S, Huber R. Chronic social stress leads to altered sleep homeostasis in mice. Behav Brain Res. 2017;327:167-73.

26.	Wells AM, Ridener E, Bourbonais CA, Kim W, Pantazopoulos H, Carroll FI, et al. Effects of Chronic Social Defeat Stress on Sleep and Circadian Rhythms Are Mitigated by Kappa-Opioid Receptor Antagonism. J Neurosci. 2017;37(32):7656-68.

27.	Basma Radwan GJaDC. Abnormal Sleep Signals Vulnerability to Chronic Social Defeat Stress. Frontiers in Neuroscience. 2020.

28.	Cho JR, Treweek JB, Robinson JE, Xiao C, Bremner LR, Greenbaum A, et al. Dorsal Raphe Dopamine Neurons Modulate Arousal and Promote Wakefulness by Salient Stimuli. Neuron. 2017;94(6):1205-19 e8.

29.	Schneeberger M, Parolari L, Das Banerjee T, Bhave V, Wang P, Patel B, et al. Regulation of Energy Expenditure by Brainstem GABA Neurons. Cell. 2019;178(3):672-85 e12.

30.	Venner A, Broadhurst RY, Sohn LT, Todd WD, Fuller PM. Selective activation of serotoninergic dorsal raphe neurons facilitates sleep through anxiolysis. Sleep. 2020;43(2).
